# Efficient separation of xylene isomers by a guest-responsive metal–organic framework with rotational anionic sites

**DOI:** 10.1038/s41467-020-19209-7

**Published:** 2020-10-28

**Authors:** Xili Cui, Zheng Niu, Chuan Shan, Lifeng Yang, Jianbo Hu, Qingju Wang, Pui Ching Lan, Yijian Li, Lukasz Wojtas, Shengqian Ma, Huabin Xing

**Affiliations:** 1 Key Laboratory of Biomass Chemical Engineering of Ministry of Education, College of Chemical and Biological Engineering, Zhejiang University, Hangzhou, 310027 China; 2 College of Chemistry, Chemical Engineering and Materials Science, Soochow University, Suzhou, 215123 China; 3 Department of Chemistry, University of South Florida, 4202 East Fowler Avenue, Tampa, Florida 33620 USA; 4 Department of Chemistry, University of North Texas, 1508W Mulberry Street, Denton, Texas 76201 USA

**Keywords:** Metal-organic frameworks, Porous materials

## Abstract

The separation of xylene isomers (*para*-, *meta*-, *orth*-) remains a great challenge in the petrochemical industry due to their similar molecular structure and physical properties. Porous materials with sensitive nanospace and selective binding sites for discriminating the subtle structural difference of isomers are urgently needed. Here, we demonstrate the adaptively molecular discrimination of xylene isomers by employing a NbOF_5_^2−^-pillared metal–organic framework (NbOFFIVE-bpy-Ni, also referred to as ZU-61) with rotational anionic sites. Single crystal X-ray diffraction studies indicate that ZU-61 with guest-responsive nanospace/sites can adapt the shape of specific isomers through geometric deformation and/or the rotation of fluorine atoms in anionic sites, thereby enabling ZU-61 to effectively differentiate xylene isomers through multiple C–H···F interactions. ZU-61 exhibited both high *meta*-xylene uptake capacity (3.4 mmol g^−1^) and *meta*-xylene/*para*-xylene separation selectivity (2.9, obtained from breakthrough curves), as well as a favorable separation sequence as confirmed by breakthrough experiments: *para*-xylene elute first with high-purity (≥99.9%), then *meta*-xylene, and *orth*-xylene. Such a remarkable performance of ZU-61 can be attributed to the type anionic binding sites together with its guest-response properties.

## Abstract (web summary)

The separation of xylene isomers remains a great challenge in industry due to their similar molecular structure and physical properties. Here the authors demonstrate adaptively molecular discrimination of xylene isomers by employing a NbOF_5_^2−^-pillared metal–organic framework with rotational anionic sites.

## Introduction

Xylene isomers are key starting materials for the production of many important chemicals and polymers, with a worldwide production exceeding 40 million metric tons per year^1,2^. Among these isomers, *para*-xylene (*p*X) is an indispensable monomer for the large-scale manufacture of poly(ethylene terephthalate) and *meta*-xylene (*m*X) is used as a fuel additive or a co-monomer for the production of high-value resins^1,3^. By 2022, the global *p*X market yield is expected to reach 66.9 billion USD. In particular, the restrictions on the purity of xylene isomers impose strict requirements for the efficient separation and purification of xylene isomers (*p*X, *m*X, *orth*-xylene (*o*-X)) and ethylbenzene (EB). However, xylene isomers have identical molecular formula and the conventional distillation method is not applicable for discerning the minor boiling point difference (138.38 °C for *p*X and 139.19 °C for *m*X)^4,5^. Currently, adsorptive separation method is an efficient alternative for the separation of xylene isomers and faujasite-type zeolites play a dominant role in industry^6^. However, traditional adsorbents suffer from low separation selectivity, which require the employment of complicated simulated moving bed technology with 24 bedlines and rotary valves^7,8^. Moreover, the intensive energy consumption for the regeneration of zeolite adsorbents represents another drawback.

**Fig. 1:**
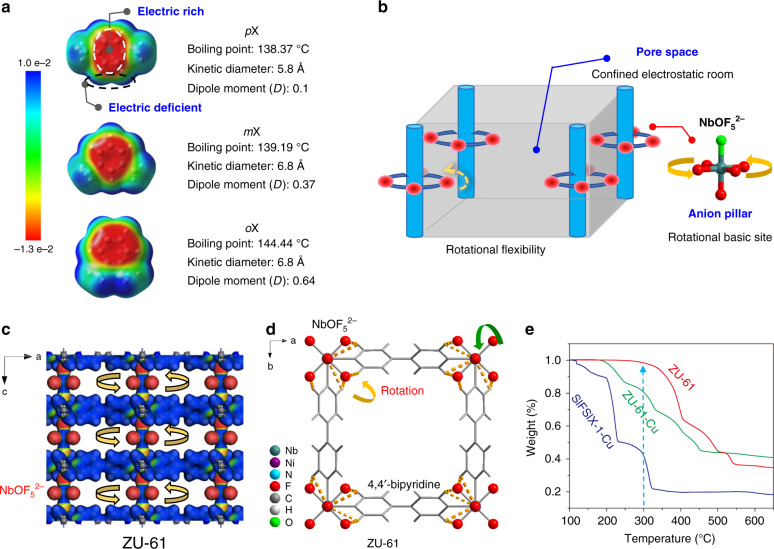
Scheme and structure of xylene isomers and ZU-61. **a** Electron density (e/au^3^) of each xylene isomers. **b** Schematic illustration of the porous adsorbent with “Lewis basic-binding sites and rotational flexibility”. **c** Structure of ZU-61 with rotational ligand of NbOF_5_^2−^ anion and bipyridine (**d**). **e** Thermogravimetric analysis results of ZU-61, SIFSIX-1-Cu, and ZU-61-Cu.

It is important and essential to understand the adsorption mechanism of xylene isomers inside the pores of the adsorbents for designing and tailoring efficient porous adsorbents. For industrial zeolitic adsorbents, investigations indicate that the strong “cation-aromatic ring” interaction between the acidic sites of the zeolite and π-electrons of the xylene isomer along with xylene packing effects determine the separation selectivity and capacity. However, these cation sites exhibited low selectivity to xylene isomers^9–12^. Moreover, the strong “cation-aromatic ring” interactions cause great energy consumption during the desorption processes. Extensive efforts have been devoted to fine-tuning of the pore size and acidity of zeolites by substituting the compensation cations. However, the separation selectivity and the capacity of zeolites are still limited by the nature of cationic binding sites and their pore topologies. Porous materials with designable pore topology and selective binding sites open up unexpected possibilities for recognition of xylene isomers^13–20^. Different kinds of metal–organic frameworks (MOFs) or porous coordination polymers, including MIL (Materials of Institut Lavoisier) series and MOFs with coordinatively unsaturated metal sites (CUSs), have been investigated for the separation of xylene isomers, both in vapor and liquid phases^21–31^. However, MIL-47 and MIL-125-NH_2_ can hardly discriminate *p*X and *m*X, and the *p*X/*m*X selectivity of MIL-47 is 1.1 (calculated from breakthrough data)^23^. For HKUST-1, the access of xylene molecules to the CUS metal center is sterically hindered, because the metal center is located at the corner of the unit cell and the *m*X/*p*X separation selectivity is 1.12 (Supplementary Table 2)^28^. Overall, the Lewis acid-binding sites (e.g., cations and CUS) in the above porous materials aim to bind with the π-electrons of the aromatic ring (Fig. 1a); however, it is difficult to discriminate xylene isomers through the interactions with aromatic ring. In addition, the traditional faujasite-type zeolites (e.g., KBaX) are *p*X-selective adsorbents, which are preferred for feedstock with low *p*X content (~20%) coming from the catalytic isomerization unit. Nowadays, with the improvement of the toluene selective disproportionation technology, the mixed feedstocks with as high as 80–95% *p*X can be easily obtained^32^. Therefore, *m*X-selective and *o*X-selective adsorbents that exhibit “inverse shape selectivity” are highly desired.

The electron density of xylene isomers (Fig. 1a) clearly shows the π-cloud of aromatic rings and the electric-positive hydrogen atoms/methyl groups. Notably, the electronic differences of the hydrogen atoms and the methyl groups in xylene isomers are more prominent than the aromatic ring, which inspired us to design a porous material with accessible electropositive/Lewis basic sites with promise to separate xylene isomers through the discrimination of the position difference of electropositive hydrogen atom in methyl groups of xylene isomers. Further, considering the similar structures of isomers, approach to maximize the discrimination ability of functional sites is requisite. In nature, dynamic molecular discrimination of the subtle structure difference by proteins is ubiquitous. Extensive structural analyses have revealed that the structure transformation of proteins enhances their ability to discriminate a specific substrate^33,34^, which inspire us to design a pore environment with certain degree of flexibility (Fig. 1b) to adapt the shape of specific guest molecules^35–38^. Thus, from a molecular point of view, porous materials with accessible Lewis basic sites together with dynamic flexibility have potential to achieve efficient separation performance for xylene isomers.

Herein, we introduced rotational ligand, NbOF_5_^2−^ anion and 4,4’-bipyridine (bpy), to construct an anion-pillared hybrid MOF, ZU-61 (ZU = Zhejiang University, also termed NbOFFIVE-bpy-Ni, NbOFFIVE = NbO_5_^2−^, bpy = 4,4’-bipyridine), which exhibits a primitive cubic (pcu) topology with square pore structures (Fig. 1c, d)^39–43^. Most importantly, the electronegative NbOF_5_^2−^ anions serving as Lewis basic sites are highly ordered and have freedom of rotation. Multi-component vapor-phase breakthrough experiments confirmed the efficient separation ability of ZU-61, with high-purity *p*X (≥99.9%) eluting first. Both the uptake capacity of *m*X (3.4 mmol/g, 333 K, and 7.1 mbar) and *m*X/*p*X separation selectivity (2.9, obtained from breakthrough curves) are higher than most top-performing MOFs and the state-of-the-art NaY zeolite. Notably, as evidenced by the crystallographic studies and calculation results, the interactions between electropositive F sites and hydrogen atoms (xylene) are enhanced by the rotation of NbOF_5_^2−^ pillars, which maximizes the discrimination ability of this material to different xylene isomers. In addition, a slight adjustment of crystal symmetry (space group) of ZU-61 was observed when accommodating *o*X.

## Results

### Synthesis and characterization of ZU-61

Anion-pillared [Ni(bpy)_2_(NbOF_5_)] (NbOFFIVE-bpy-Ni, also termed ZU-61) and [Cu(bpy)_2_(NbOF_5_)] (NbOFFIVE-bpy-Cu, also termed ZU-61-Cu) were prepared by the reaction of bpy and NiNbOF_5_, and CuNbOF_5_, respectively. ZU-61, as an isomorph of SIFSIX-1-Cu^39,40^, exhibit three-dimensional framework with pcu topology. The one-dimensional channels of ZU-61 are aligned by a high density of rotational anion pillars (Fig. 1c, d). The four uncoordinated fluoride atoms of NbOF_5_^2−^ anion extend out toward the channel, behaving as highly accessible adsorption sites. The bulk purity of the anion-pillared MOF samples was confirmed by the powder X-ray diffraction (PXRD) tests (Supplementary Fig. 1). The pore size distribution of ZU-61 is centered at 7.8 Å and its surface area is 1384 m^2^/g (Supplementary Figs. 3 and 4). It is worth noting that NbOFFIVE-bpy-Ni exhibits greatly improved thermal stability than its analog of SIFSIX-1-Cu and NbOFFIVE-bpy-Cu, as confirmed by the thermogravimetric analysis tests (Fig. 1e). At 300 °C, NbOFFIVE-bpy-Ni lost only 1.58% weight, whereas SIFSIX-1-Cu lost 58% weight. NbOFFIVE-bpy-Ni also displayed higher stability towards humidity in comparison with SIFSIX-1-Cu, as shown in the water adsorption–desorption tests (Supplementary Fig. 5). This high thermal and water vapor stability of NbOFFIVE-bpy-Ni is beneficial to its potential industrial applications.

### Breakthrough separation of ternary mixture of *p*X, *m*X, *o*X

**Fig. 2:**
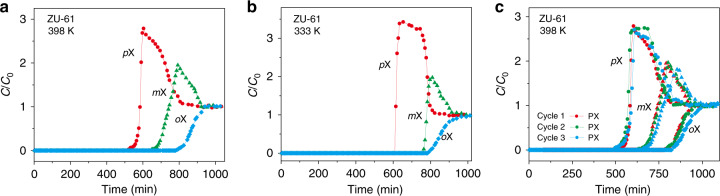
Experimental column breakthrough results of ZU-61. Breakthrough curves for 1:1:1 *p*X/*m*X/*o*X separations with ZU-61 at 398 K (**a**), 333 K (**b**), and recycle performance at 398 K (**c**).

Breakthrough tests are efficient tools to screen suitable adsorbents. The separation ability of adsorbents could be straightforwardly evaluated with the dynamic multi-component breakthrough experiments in vapor phase. The sequence and the shape of each xylene isomer breakthrough curve is good means to elucidate the separation mechanisms. In this work, we used a *m*X-selective zeolite NaY (pore aperture of 7.4 Å, surface area of 880 m^2^/g) and *p*X-selective zeolite BaY as the reference adsorbent. We evaluated the separation performance of ZU-61 and zeolite NaY and BaY for the mixed isomers of *p*X, *m*X, *o*X in the gas phase (1:1:1 in nitrogen). Excellent separation performance was achieved on ZU-61 column at 398 K (Fig. 2a). Pure *p*X eluted with a marked roll-up, followed by *m*X, and then *o*X sequentially. The separation selectivity for *m*X/*p*X and *o*X/*p*X is 1.9 and 2.6, respectively (obtained from breakthrough curves). For comparison, zeolite NaY, tested under the same condition as ZU-61, showed poorer separation performance (Supplementary Fig. 7). As for the breakthrough results of zeolite BaY (Supplementary Fig. 11), *m*X eluted first and followed by *o*X, and then *p*X sequentially. The breakthrough sequence of xylene isomers tested on BaY confirmed the excellent performance with *p*X as the minority component in the feed. The close breakthrough time of *p*X, *o*X, and *m*X on zeolite NaY indicate that zeolite NaY hardly discriminate *p*X, *o*X, and *m*X at this condition. The breakthrough sequence of the xylene isomers tested on ZU-61 at lower temperature of 333 K followed the same order of that performed at 398 K (Fig. 2b). Notably, the separation selectivity of *m*X/*p*X was increased from 1.9 (at 398 K) to 2.9 (at 333 K). This value is higher than HKUST-1 (1.12 at 398 K)^28^, calculated from binary breakthrough experiments conducted at 398 K (Supplementary Table 2). Before the elution of *m*X, the purity of *p*X is >99.9%. Given that *p*X is the most desirable chemical in industry, the phenomena that *p*X elutes first from the ZU-61 column can increase the purity of *p*X and may facilitate to cut the total energy consumption of *p*X production, especially for the industrial separation unit employing 80–95% *p*X mixtures as feedstock. Therefore, ZU-61 would be favored for possible industrial applications. The marked roll-up of *p*X indicated that the weakly adsorbed *p*X was partially desorbed by the subsequent strongly adsorbed *m*X and *o*X. In addition, the liquid-phase multi-component breakthrough experiments (Supplementary Fig. 13) were performed on the column of ZU-61. The results confirmed that *p*X is the least adsorbed isomer (Supplementary Fig. 14). By washing the column of ZU-61 with *para*-diethylbenzene (PDEB), the adsorbed xylene isomers can be desorbed by PDEB. And on the next cycle, xylene isomers could displace PDEB.

Considering the coexistence of EB, we also conducted the breakthrough experiments using an equimolar mixture of *p*X, *m*X, *o*X, and EB in nitrogen. Results (Supplementary Fig. 6) showed that *p*X elute first from the column of ZU-61 at 398 K and the separation selectivity of ZU-61 for *m*X/*p*X and *o*X/*p*X were almost unaffected by the presence of EB. Because of the collapse of pore structure of SIFSIX-1-Cu at 373 K, we only tested the performance of SIFSIX-1-Cu at a lower temperature of 333 K (Supplementary Fig. 8). In addition, the regeneration conditions and the cycle performance of ZU-61 were systematically investigated, and the results indicated that ZU-61 showed good recyclability (Fig. 2c). Regeneration tests showed that the desorption of xylene isomers on ZU-61 (423 K for 12 h) needed lower temperature and shorter time than zeolite NaY (533 K for 20 h), suggesting ZU-61 was more energy efficient.

### Single-component adsorption isotherms of xylene isomer

**Fig. 3:**
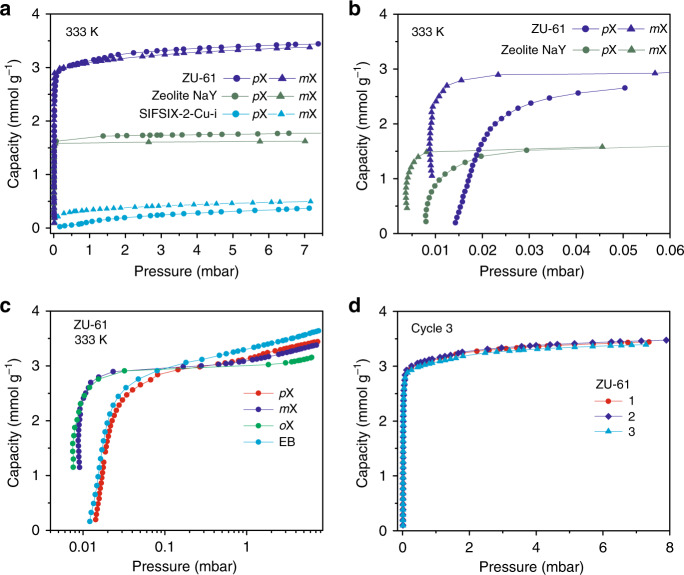
Single-component adsorption properties of ZU-61 and Zeolite NaY. Adsorption isotherms of *p*X and *m*X on ZU-61, SIFSIX-2-Cu-i, and zeolite NaY at 333 K in two pressure regions, 0–7 mbar (**a**) and 0–0.06 mbar (**b**). The comparison of *p*X, *m*X, *o*X, and EB adsorption behavior on ZU-61 (**c**) and cycle adsorption of *p*X on ZU-61 (**d**) at 333 K.

To further investigate the thermodynamic adsorption properties, single-component adsorption isotherms of *p*X, *m*X, *o*X, and EB on ZU-61, SIFSIX-2-Cu-i, and zeolite NaY and BaY were measured at the temperatures of 298 and 333 K. ZU-61, zeolite NaY and BaY all showed steep increase at ultra-low pressure and reached saturation at about 0.03 mbar (Fig. 3a). In comparison, SIFSIX-2-Cu-i had very low uptake of *p*X and *m*X molecules, as a result of the small pore size of SIFSIX-2-Cu-i (5.15 Å). At the plateau of the isotherms, the *p*X and *m*X adsorption capacity of ZU-61 was 3.44 and 3.37 mmol/g (7 mbar and 333 K), respectively, almost twice the amount of the *p*X (1.77 mmol/g) and *m*X (1.62 mmol/g) by zeolite NaY. The *p*X and *m*X adsorption capacity of BaY was 2.47 and 2.51 mmol/g (7 mbar and 298 K; Supplementary Fig. 12), respectively, higher than the capacity of NaY. The higher adsorption capacity of ZU-61 than zeolite NaY is consistent with the breakthrough experiments. Therefore, ZU-61 not only exhibited excellent separation selectivity for xylene isomers but also possessed high *m*X capacity, which renders ZU-61 a more promising adsorbent for the *p*X purification. To obtain the accurate adsorption enthalpy, we employed the thermogravimetric-differential scanning calorimetry (DSC) to measure the adsorption enthalpy of each isomer (Supplementary Fig. 19). The adsorption enthalpy of *p*X, *m*X, and *o*X for ZU-61 were 78, 82, 92 kJ/mol, respectively.

To investigate the subtle xylene adsorption difference on ZU-61 and zeolite NaY, adsorption isotherms of *p*X and *m*X under low-pressure range (0–0.06 mbar) were compared (Fig. 3b). For ultra-low pressure below 0.01 mbar, zeolite NaY showed stronger adsorption of *p*X and *m*X than ZU-61. The adsorption isotherm of *m*X on zeolite NaY was very steep at the lower pressure range and reached saturation at an ultra-low pressure of 0.01 mbar and 333 K. The strong binding of xylene isomers within zeolite NaY was consistent with its demanding regeneration condition. Furthermore, the isotherms of *m*X are significantly steeper than those of *p*X on ZU-61 at the low-pressure range 0–0.05 mbar, showing these adsorbents are *m*X-selective adsorbents (Fig. 3b). A direct comparison of the adsorption isotherms of four C_8_ aromatics (*p*X, *m*X, *o*X, and EB) on ZU-61 shows that at low-pressure range of 0–0.1 mbar, the adsorption preference of ZU-61 towards the four isomers followed the order of *o*X > *m*X > EB > *p*X (Fig. 3c). This static adsorption behaviors of ZU-61 were consistent with the results of breakthrough tests. In addition, static multiple adsorption–desorption experiments also indicated the excellent cycle performance of ZU-61 for *p*X adsorption (Fig. 3d).

### Determination of xylene adsorption sites in ZU-61

**Fig. 4:**
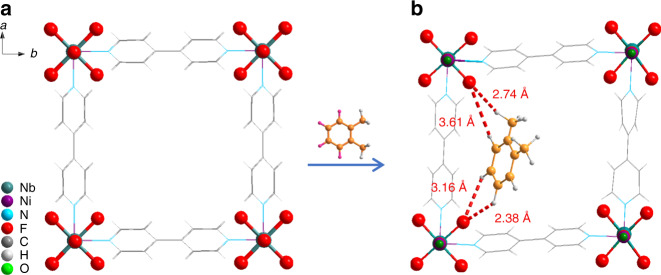
Single-crystal X-ray diffraction resolved structure of ZU-61·*o*X. Crystal structure of ZU-61 before (**a**) and after adsorption of *o*-xylene ZU-61·*o*X (**b**). Color code: C, gray 50%; H, gray 10%; Nb, teal; Ni, violet; O, green; N, sky blue; F, red. Note: for the structure of ZU-61·*o*X, the single-crystal diffraction data are used to resolve the structure of ZU-61 and locate the position of benzene ring of *o*X, additional DFT-D calculations are employed to locate the position of methyl groups of *o*X.

**Fig. 5:**
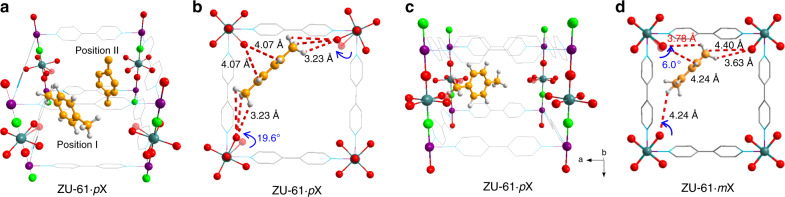
Single-crystal X-ray diffraction resolved structure of ZU-61·*p*X and ZU-61·*m*X. Two adsorption positions of *p*X molecules in the unite cell of ZU-61 (**a**). ZU-61·*p*X with *p*X molecule in Position I in different directions (**b**, **c**). Location of *m*X in the pores of ZU-61 (**d**). Color code: C, gray 50%; H, gray 10%; Nb, teal; Ni, violet; O, green; N, sky blue; F, red. Note: the single-crystal diffraction data are not of sufficient quality to locate the precise positions of methyl groups of *m*X in ZU-61·*m*X. Therefore, we just give the adsorption positions of benzene ring, additional DFT-D calculations are employed to locate the position of methyl groups of *m*X.

Single-crystal X-ray diffraction studies were performed to locate the adsorption sites of xylene in the channel of anion-pillared MOFs. Remarkably, to fit the strong interactions with *o*X, ZU-61 was found to undergo a structural deformation (Fig. 4). The unit cell parameters of ZU-61 (*a* = *b* = 11.26, *c* = 7.89; Fig. 4a) transformed into (*a* = 22.48, *b* = 7.86, *c* = 11.25; Fig. 4b) ZU-61^p^ (we refer the *o*X-adsorbed ZU-61 structure as ZU-61^p^). After adsorption of *o*X, the original square geometry of the Cu-bpy framework transformed into the parallelogram. The adsorption behavior of *o*X is dramatically different from *p*X (Fig. 5). As the single-crystal X-ray diffraction data can only locate the benzene ring of *o*X, additional dispersion-corrected density functional theory (DFT-D) calculations were used to determine the position of methyl groups of *o*X (Supplementary Information). The DFT-D-calculated static binding energy of *o*X is as high as 109.8 kJ/mol. As shown in Fig. 4b, the synergetic C–H···F interactions along with *o*-xylene_benzene_–bipy_benzene_ interactions enabled the strong binding of *o*X in ZU-61. There are two kinds of hydrogen in xylene molecules: aromatic hydrogen (H_aryl_) that binds to the benzene ring and methyl hydrogen (H_benzyl_). The distance between H_aryl_ and F atoms were 2.38, 3.16, and 3.61 Å, respectively. The distance between H_benzyl_ and F atoms were 2.74 and 3.75 Å, respectively. These synergetic C–H···F interactions enable the strongest energy of *o*X binding. In contrast, the centroid-to-centroid distances of *o*-xylene_benzene_–bipy_benzene_ ranged from 5.00 to 5.44 Å, indicating that the arene *π*–*π* interactions between xylene and bipyridine were relatively weak. Compared with the interaction of ZU-61 with *p*X, the strong host–guest interactions with *o*X resulted in the transformation of the framework. Therefore, the crystallographic studies demonstrated that ZU-61 can adjust its geometry to discriminate each xylene isomers based on their shape difference (methyl position difference). This self-adjustment properties of ZU-61 enable the discrimination of isomers with subtle differences.

As for the adsorption of *p*X, the single-crystal X-ray diffraction data of ZU-61·*p*X indicate that there are two adsorption positions of *p*X molecules in the pores of ZU-61 (Fig. 5a) and there is no structural deformation in the framework of ZU-61. As shown in Fig. 5a, these two positions of *p*X are named as Position I and Position II. Notably, an obvious rotation of uncoordinated F atoms (19.6°) can be visualized by diffraction data. When adsorbed at Position I, one *p*X molecule binds with three F atoms by synergetic C–H···F interactions between hydrogen in xylene molecules and electronegative F atoms (Fig. 5b). The two methyl groups of *p*X interact with two F atoms in the diagonal position within the unit cell of ZU-61. The distance between H_benzyl_ and F atoms are ranged from 3.23 to 4.33 Å. The four H_aryl_ interact with the F atoms and the distance between H_aryl_ and F atoms are 4.07 Å. As shown in Fig. 5c, it should be noted that the *p*X molecule is trapped by the anion sites and *p*X cannot form *π*–*π* interactions with the aromatic ring of bipyridine. Therefore, multiple C–H···F interactions between electronegative F atoms and hydrogen in *p*X molecule play a more critical role than the *π*–*π* interactions. Further, the location of *p*X molecules at Position II is perpendicular to those at Position I, forming optimal packing of two *p*X molecules. The single-crystal X-ray diffraction data were also collected for *m*X adsorbed on ZU-61 (Fig. 5d). Crystallographic information showed that the framework of ZU-61 did not undergo a structural deformation, while a rotation (6°) of uncoordinated F sites to adapt the shape of *m*X is detected. DFT-D calculations were performed to locate the position of methyl groups, whereas crystallographic data were used to locate the benzene ring of *m*X. One *m*X molecule interact with three F atoms by synergetic C–H···F interactions between hydrogen in xylene molecules and electronegative F atoms (Fig. 5d). The distance between H_benzyl_ and F atoms are ranged from 3.63 to 4.97 Å, and the distance between H_aryl_ and F atoms are ranged from 4.24 to 4.48 Å. Besides, single-crystal characterizations indicated that the organic ligands of bipyridine would rotate to adapt the adsorption of xylene molecules (Supplementary Fig. 17). In the unit cell of the resolved structure of ZU-61 without xylene molecules, the dihedral angle is 34.06°. Then after the adsorption of *p*X and *m*X molecules, the dihedral angle changed to 26.17° and 25.86°, respectively. As revealed by these results, the smaller dihedral angle of 25.86° indicate the larger pore when *m*X adsorbed. Overall, for the adsorption of *p*X and *m*X, basic anions are the main binding sites and the rotation of uncoordinated F atoms provides optimal interactions for guest molecules in response to the shape difference of xylene isomers.

## Discussion

In summary, the efficient separation of xylene isomers has been achieved through a rationally designed adsorbent of ZU-61 featuring adaptable pore structure with accessible and rotational Lewis basic sites, as well as excellent thermal and humidity stability. Both static single-component adsorption isotherms and dynamic multi-component breakthrough experiments confirmed the preferential adsorption sequence of ZU-61: *o*X > *m*X > *p*X, which is especially preferred in the industrial separation unit employing 80–95% *p*X mixtures as feedstock. With this optimal adsorption sequence, a high-purity *p*X (≥99.9%) could be directly obtained by a one-step fixed-bed adsorption without using complicated processes. In addition, the ZU-61 exhibited both high uptake capacity for *o*X and *m*X (3.2 and 3.4 mmol/g at 333 K and 7.1 mbar) and high separation selectivity for *m*X over *p*X (2.9, obtained from breakthrough curves), which are superior to state-of-the-art zeolites and MOFs. The easy regeneration of ZU-61 could circumvent the high energy consumption as required for zeolite NaY. Crystallographic studies indicated that electropositive/basic NbOF_5_^2−^ anions are main sites for biding xylene isomers through C–H···F interactions. More important, ZU-61 as a guest-responsive porous material could adapt the shape of different xylene isomers through structural deformation and/or the rotation of NbOF_5_^2−^ anions, which enhances the discrimination ability of ZU-61 for structurally similar isomers. Therefore, we attribute this remarkable performance to a type Lewis basic anionic-binding site for xylene isomers. This binding site presents a paradigm shift compared traditional Lewis acidic sites found in previous materials. This work not only paves a path forward for industrial *p*X separation and purification, but also advances a broadly approach applicable to other important isomer separations.

## Methods

### Synthesis of ZU-61 (Ni(4,4’-bipyridylacetylene)_2_NbOF_5_)_*n*_

Bpy (0.35 g) was dissolved in 40 mL ethylene glycol at 65 °C. An aqueous solution (20 mL) of NiNbOF_5_ (0.41 g) was added to the above solution. Then the mixture was heated at 65 °C for 1 h under stirring. The obtained powder was filtered, washed with methanol.

### Synthesis of ZU-61-Cu (Cu(4,4’-bipyridylacetylene)_2_NbOF_5_)_*n*_

Bpy (0.35 g) was dissolved in 40 mL ethylene glycol at 65 °C. An aqueous solution (20 mL) of NiNbOF_5_ (0.39 g) was added to the above solution. Then the mixture was heated at 65 °C for 1 h under stirring. The obtained powder was filtered, washed with methanol.

### Synthesis of SIFSIX-1-Cu (Cu(4,4’-bipyridine)_2_SiF_6_)_*n*_

Bpy (0.35 g) was dissolved in 40 mL ethylene glycol at 338 K. An aqueous solution (20 mL) of Cu(BF_4_)_2_•xH_2_O (266 mg, 1.12 mmol) and (NH_4_)_2_SiF_6_ (199 mg, 1.12 mmol) was added to the above solution. Then the mixture was heated at 65 °C for 3 h under stirring. The obtained purple powder was filtered, washed with methanol, and then exchanged with methanol for 3 days.

### Preparation of xylene loaded ZU-61

The crystals of ZU-61 were degassed at 333 K until the pressure dropped below 10 μm Hg. Then the vapor of pure xylene was introduced into the sample of activated ZU-61 until the pressure reach to 7 mbar at 298 K. After 2 h, the crystals were covered with the degassed oil in the glove box for single-crystal X-ray diffraction tests.

### Single-crystal X-ray diffraction structure analysis

Crystal data for ZU-61 and other samples were collected on a Bruker D8 Venture PHOTON II CPAD system equipped with a Cu-Kα INCOATEC ImuS micro-focus source (*λ* = 1.54178 Å). Indexing was performed using APEX3 (Difference Vectors method). Data integration and reduction were performed using SaintPlus. Absorption correction was performed by multi-scan method implemented in SADABS. Space groups were determined using XPREP implemented in APEX3. Structures were solved using SHELXT and refined using SHELXL-2016 (full-matrix leastsquares on F^2^) through OLEX2 interface program.

### PXRD structure analysis

PXRD was carried out at room temperature on a Bruker D8 Advance diffractometer using Cu-Kα radiation (*λ* = 1.5418 Å).

### C_8_ aromatics vapor adsorption

ZU-61, SIFSIX-1-Cu, SIFSIX-2-Cu-i, and zeolite NaY were degassed at certain temperature until the pressure dropped below 10μm Hg. Nitrogen adsorption–desorption isotherms at 77 K were collected using ASAP 2020 Analyzer (Micromeritics). The single-component vapor adsorption isotherm of the activated samples were collected using ASAP 2020 Analyzer equipped with a vapor dosing tube. Each xylene isomers and ethylbeneze was purified by being degassed on ASAP 2020 through freeze–pump–thaw cycles.

### Vapor-phase breakthrough tests

The vapor-phase multi-component breakthrough tests were carried out in a dynamic vapor breakthrough equipment. All experiments were conducted using a stainless-steel column (4.6 mm inner diameter × 50 mm). The column packed with adsorbent was first purged with He flow at room temperature. The mixed gases of *p*X, *m*X, *o*X (1:1:1) and *p*X, *m*X, *o*X, EB (1:1:1:1) in nitrogen were produced by nitrogen-blow bubble method. Nitrogen was passed through the container of C_8_ aromatics liquid mixture at desired rate. After the concentration of each isomer were tuned to the desired value, introduce the mixed gas in nitrogen at 25 mL/min. Outlet gas from the column was monitored using gas chromatography (GC) (GC-2010, SHIMADZU). The vapor mixture was separated by a capillary column (Agilent). It should be note that the time caused by the void volume of the pipeline and the column have been deducted when processing the breakthrough data.

To control and calculate the component ratios of the mixture, specific procedures were performed as follows: (1) before performing the breakthrough experiments, we first measured and got the Relative Quality Correction Factor of *p*X, *m*X, *o*X, and EB (Supplementary Table 5) on our GC with Flame Ionization Detector (FID). (2) According to the Antoine equation, the saturated pressure of each xylene isomer and EB can be calculated (Supplementary Table 6). Then, the vapor of xylene mixtures with known liquid compositions were injected and measured on the GC. Therefore, the corresponding relationship between each xylene isomer concentration and the response value of FID detector were obtained (Supplementary Table 7). Based on the above information, we can test the concentration/pressure of respective component. (3) For the vapor-phase breakthrough tests, the mixed vapors of *p*X, *m*X, *o*X (1:1:1) in nitrogen were produced by nitrogen-blow bubble method. Nitrogen was passed through the container of C_8_ aromatics liquid mixture at desired rate and injected into GC to measure the concentration. According to the tested concentrations, adjust the liquid mixtures until the gas concentration reached ~1:1:1.

### The Antoine equation

The saturated pressure of *p*X, *m*X, *o*X, and EB

1 $${\mathrm{log}}P = A - \frac{B}{{t + C}}$$

Here, *P* is the pressure expressed in mm Hg. *A*, *B*, and *C* are constants and the physical property data can be found in various manuals. *T* is the temperature expressed in °C. The *A*, *B*, *C* values of *p*X, *m*X, *o*X, and EB are provided in the Supplementary Table 6.

*Liquid-phase breakthrough tests*: The liquid-phase multi-component breakthrough tests were carried out in a liquid breakthrough equipment. All experiments were conducted using a stainless-steel column (4.6 mm inner diameter × 100 mm). The liquid mixture of *p*X, *m*X, and *o*X (1:1:1) was diluted with heptane or hexane, the concentration of each xylene isomer is 0.01 mmol/mL. The fluid is pumped by the high-performance liquid chromatography pump with flow rate of 0.2 ml/min. The concentration of effluent was detected using GC with FID detector. Furthermore, the column of ZU-61 was washed by PDEB (diluted with heptane) and then on the next cycle the liquid mixture of *p*X, *m*X, and *o*X (1:1:1, diluted with heptane) was pumped into the column. In addition, we evaluated the separation performance of ZU-61 for binary *p*X/*m*X mixtures using the liquid-phase breakthrough equipment.

*Differential scanning calorimetry*: Enthalpy of adsorption for xylene isomers was measured using the PE TGA and PE DSC 7. ZU-61 and zeolite NaY were activated on the PE TGA at certain temperatures under dry N_2_ flow until the weights remained stable. The activated samples were transferred to the PE DSC 7, to measure the adsorption enthalpy, the baseline was obtained under dry N_2_ flow at 25 °C, then the N_2_ was changed to xylene isomers and the DSC signal were monitored to the obtain the heat of adsorption.

### DFT calculations

The DFT calculations were performed to calculate the static binding energy using the CASTEP code. A semi-empirical addition of dispersive forces to conventional DFT was included in the calculation to account for van der Waals interactions. We used Vanderbilt-type ultrasoft pseudopotentials and generalized gradient approximation (GGA) with Perdew–Burke–Ernzerhof (PBE) exchange correlation. A cutoff energy of 544 ev and a 1 × 1 × 2 *k*-point mesh (generated using the Monkhosrt–Pack scheme) were found to be enough for the total energy to converge within 0.01 meV/atom. The structure of ZU-61 was first optimized. The optimized structures are good matches for the experimentally determined crystal structures of the coordination networks. The PX molecules were then introduced into the optimized structure, followed by a full structural relaxation. The initial location of *p*X molecules were obtained from the experiment XRD data. To obtain the gas binding energy, an isolated gas molecule placed in a supercell (with the same cell dimensions as the MOF crystal) was also relaxed as a reference. The static binding energy (at *T* = 0 K) was then calculated using: EB = E(MOF) + E(gas) − E(MOF + gas).

To confirm the adsorption configuration of *o*X and *m*X in ZU-61, the first-principle DFT and plane-wave ultrasoft pseudopotentil implemented in the CASTEP code were performed. First, six possible adsorption configurations of *o*X/*m*X in the structure of ZU-61 were constructed based on the experimental single-crystal X-ray diffraction data. Then, the single point energy calculations were performed to calculate the energy of these six possible configurations. All of the calculations were performed under the GGA with PBE exchange correlation. The cutoff energy of 544 eV and 1 × 1 × 2 *k*-point mesh with smearing 0.1 ev were adopted in the calculation. Finally, the energy of these configurations was compared to confirm the stable *o*X/*m*X configuration in ZU-61 structure (Supplementary Table 4). The higher negative value of single point energy corresponds to a more stable adsorption configuration.

## Supplementary information

Supplementary Information

## Data Availability

The data that support the findings of this study are available from the corresponding author upon reasonable request. Correspondence and requests for materials should be addressed to X.C., S.M., and H.X. CCDC 1982509, 1982972, and 1982976 entries contain supplementary single-crystal X-ray diffraction data for ZU-61 and xylene adsorbed structures. These data can be obtained free of charge from The Cambridge Crystallographic Data Centre via https://www.ccdc.cam.ac.uk/structures/.
