# Supplementary material for: Efficient separation of xylene isomers by a guest-responsive metal–organic framework with rotational anionic sites
Source: Nat Commun. 2020 Oct 28;11:5456. doi: 10.1038/s41467-020-19209-7 (PMC7595167; doi:10.1038/s41467-020-19209-7)
Supplement: Supplementary file 1 — Supplementary Information [file 41467_2020_19209_MOESM1_ESM.pdf]

Supplementary Information for:

Efficient Separation of Xylene Isomers by A Guest-Responsive Metal-organic Framework with Rotational Anionic Sites

Xili Cui[1]*, Zheng Niu[2], Chuan Shan[3], Lifeng Yang[1], Jianbo Hu[1], Qingju Wang[1], Pui Ching Lan[4], Yijian Li[1], Lukasz Wojtas[3], Shengqian Ma[4]*, Huabin Xing[1]*

[1] Key Laboratory of Biomass Chemical Engineering of Ministry of Education, College of Chemical and Biological Engineering, Zhejiang University, Hangzhou 310027, China.

[2] College of Chemistry, Chemical Engineering and Materials Science, Soochow University, Suzhou 215123, P. R. China

[3] Department of Chemistry, University of South Florida, 4202 East Fowler Avenue, Tampa, Florida 33620, USA

[4] Department of Chemistry, University of North Texas, 1508 W Mulberry St, Denton, TX 76201, USA

*To whom correspondence should be addressed. E-mails: cuixl@zju.edu.cn (X.C.); Shengqian.Ma@unt.edu (S.M.); xinghb@zju.edu.cn (H.X.)

Experiment

Materials

$NiNbOF_5$ (98%), ammonium hexafluorosilicate ($(NH)_2SiF_6$, 98%, Aldrich), copper (II) tetrafluoroborate hydrate ($Cu(BF_4)_2$• xH_2O, 98%, Aldrich), 4,4'-bipyridine ($C_{10}H_8N_2$, 98%, Aldrich), 4-(2-pyridin-4-ylethynyl)pyridine ($C_{12}H_8N_2$, 98%, Chemsoon), pyrazine ($C_4H_4N_2$, 99%, Aldrich), methanol (CH_3OH, anhydrous, 99%, Sigma-Aldrich), ethylene glycol ($C_2H_6O_2$, anhydrous, 99%, Sigma-Aldrich), were purchased and used without further purification.

N_2 (99.999%), He (99.999%) were purchased form Airgas. Mixed gases of *p*X, *m*X, *o*X (1:1:1) in nitrogen were produced by flowing nitrogen through the bubbler of xylene mixture. The composition of xylene mixture in the vapor phase were adjusted by the composition in the liquid phase of xylene mixture.

Synthesis

Synthesis of ZU-61 (Ni(4,4'-bipyridylacetylene)$_2$NbOF$_5$)n

0.35 g 4,4'-bipyridine was dissolved in 40 mL ethylene glycol at 65 °C. An aqueous solution (20 mL) of $NiNbOF_5$ (0.41 g) was added to the above solution. Then the mixture was heated at 65 °C for 1 h under stirring. The obtained powder was filtered, washed with methanol.

Synthesis of ZU-61-Cu (Cu(4,4'-bipyridylacetylene)$_2$NbOF$_5$)n

0.35 g 4,4'-bipyridine was dissolved in 40 mL ethylene glycol at 65 °C. An aqueous solution (20 mL) of $NiNbOF_5$ (0.39 g) was added to the above solution. Then the mixture was heated at 65 °C for 1 h under stirring. The obtained powder was filtered, washed with methanol.

Synthesis of SIFSIX-1-Cu (Cu(4,4'-bipyridine)$_2$SiF$_6$•8H$_2$O)n

Firstly, 0.35 g 4,4'-bipyridine was dissolved in 40 mL ethylene glycol at 338 K. An aqueous solution (20 mL) of $Cu(BF_4)_2$•xH_2O (266 mg, 1.12 mmol) and $(NH_4)_2SiF_6$ (199 mg, 1.12 mmol) was added to the above solution. Then the mixture was heated at 65 °C for 3 h under stirring. The obtained purple powder was filtered, washed with methanol, and was exchanged with methanol for 3 days.

Preparation of xylene loaded ZU-61

The crystals of ZU-61 were degassed at 333 K until the pressure dropped below 10μm Hg. Then the vapor of pure xylene was introduced into the sample of activated ZU-61 until the pressure reach to 7 mbar at 298 K. After 2 hours, the crystals were covered with the degassed oil in the glove box for single crystal X-ray diffraction tests.

Single-crystal X-ray diffraction structure analysis

Crystal data for ZU-61 and other samples were collected on a Bruker D8 Venture PHOTON II CPAD system equipped with a Cu Kα INCOATEC ImuS micro-focus source (λ = 1.54178 Å). Indexing was performed using APEX3[1] (Difference Vectors method). Data integration and reduction were performed using SaintPlus[2]. Absorption correction was performed by multi-scan method implemented in SADABS[3]. Space groups were determined using XPREP implemented in APEX3[1]. Structures were

solved using SHELXT and refined using SHELXL-2016[3-7] (full-matrix leastsquares on F^2) through OLEX2 interface program[8].

Powder X-ray diffraction structure analysis

Powder X-ray diffraction (PXRD) was carried out at room temperature on a Bruker D8 Advance diffractometer using Cu-Kα radiation (λ=1.5418 Å).

C_8 aromatics vapor adsorption

ZU-61, SIFSIX-1-Cu, SIFSIX-2-Cu-i, and zeolite NaY were degassed at certain temperature until the pressure dropped below 10μm Hg. Nitrogen adsorption-desorption isotherms at 77 K were collected using ASAP 2020 Analyzer (Micromeritics). The single-component vapor adsorption isotherm of the activated samples were collected using ASAP 2020 Analyzer equipped with a vapor dosing tube. Each xylene isomers and ethylbeneze was purified by being degassed on ASAP 2020 through freeze-pump-thaw cycles.

Vapor-phase breakthrough tests

The vapor-phase multi-component breakthrough tests were carried out in a dynamic vapor breakthrough equipment. All experiments were conducted using a stainless-steel column (4.6 mm inner diameter × 50 mm). The column packed with adsorbent was firstly purged with He flow at room temperature. The mixed gases of *p*X, *m*X, *o*X (1:1:1) and *p*X, *m*X, *o*X, EB (1:1:1:1) in nitrogen were produced by nitrogen-blow bubble method. Nitrogen was passed through the container of C_8 aromatics liquid mixture at desired rate. After the concentration of each isomer were tuned to the desired value, introduce the mixed gas in nitrogen at 25 mL/min. Outlet gas from the column was monitored using gas chromatography (GC-2010, SHIMADZU). The vapor mixture was separated by a capillary column (Agilent). It should be note that the time caused by the void volume of the pipeline and the column have been deducted when processing the breakthrough data.

To control and calculate the component ratios of the mixture, specific procedures were performed: 1) Before performing the breakthrough experiments, we firstly measured and got the Relative Quality Correction Factor of *p*X, *m*X, *o*X, and EB (Supplementary Table 5) on our gas chromatography (GC) with Flame Ionization Detector (FID). 2) According to the Antoine equation, the saturated pressure of each xylene isomer and EB can be calculated (Supplementary Table 6). Then, the vapor of xylene mixtures with known liquid compositions were injected and measured on the GC. Therefore, the corresponding relationship between each xylene isomer concentration and the response value of FID detector were obtained (Supplementary Table 7). Based on the above information, we can test the concentration/pressure of respective component. 3) For the vapor-phase breakthrough tests, the mixed vapors of *p*X, *m*X, *o*X (1:1:1) in nitrogen were produced by nitrogen-blow bubble method. Nitrogen was passed through the container of C8 aromatics liquid mixture at desired rate and injected into GC to measure the concentration. According to the tested concentrations, adjust the liquid mixtures until the gas concentration reached approximately 1:1:1.

The Antoine equation

The saturated pressure of pX, mX, oX and ethylbenzene

$$logP = A - \frac{B}{t+C} \quad (1)$$

Here, P is the pressure expressed in mmHg. A, B and C is a constant and the physical property data can be found in various manuals. T is the temperature expressed in ℃. The A, B, C values of pX, mX, oX and ethylbenzene are provided in the Supplementary Table 6.

Liquid-phase breakthrough tests

The liquid-phase multi-component breakthrough tests were carried out in a liquid breakthrough equipment. All experiments were conducted using a stainless-steel column (4.6 mm inner diameter × 100 mm). The liquid mixture of pX, mX, and oX (1:1:1) was diluted with heptane or hexane, the concentration of each xylene isomer is 0.01 mmol/mL. The fluid is pumped by the HPLC pump with flow rate of 0.2 ml/min. The concentration of effluent was detected using GC with FID detector. Furthermore, the column of ZU-61 was washed by para-diethylbenzene (diluted with heptane), and then on the next cycle the liquid mixture of pX, mX, and oX (1:1:1, diluted with heptane) was pumped into the column. In addition, we evaluated the separation performance of ZU-61 for binary pX/mX mixtures using the liquid-phase breakthrough equipment.

Differential Scanning Calorimetry (DSC)

Enthalpy of adsorption for xylene isomers was measured using the PE TGA and PE DSC 7. ZU-61 and zeolite NaY were activated on the PE TGA at certain temperatures under dry N_2 flow until the weights remained stable. The activated samples were transferred to the PE DSC 7 to measure the adsorption enthalpy, the baseline was obtained under dry N_2 flow at 25°C, then the N_2 was changed to xylene isomers and the DSC signal were monitored to the obtain the heat of adsorption.

Density-functional theory calculations

The density-functional theory (DFT) calculations were performed to calculate the static binding energy using the CASTEP code. A semi-empirical addition of dispersive forces to conventional DFT was included in the calculation to account for van der Waals interactions. We used Vanderbilt-type ultrasoft pseudopotentials and generalized gradient approximation (GGA) with Perdew-Burke-Ernzerhof (PBE) exchange correlation. A cutoff energy of 544 ev and a 1×1×2 k-point mesh (generated using the Monkhosrt-Pack scheme) were found to be enough for the total energy to converge within 0.01 meV/atom. The structure of ZU-61 was first optimized. The optimized structures are good matches for the experimentally determined crystal structures of the coordination networks. The PX molecules were then introduced into the optimized structure, followed by a full structural relaxation. The initial location of pX molecules were obtained from the experiment XRD data. To obtain the gas binding energy, an isolated gas molecule placed in a

supercell (with the same cell dimensions as the MOF crystal) was also relaxed as a reference. The static binding energy (at T = 0 K) was then calculated using: EB = E(MOF) + E(gas) – E(MOF+gas).

In order to confirm the adsorption configuration of *o*X and *m*X in ZU-61, the first-principle density functional theory (DFT) and plane-wave ultrasoft pseudopotentil implemented in the CASTEP code were performed. Firstly, six possible adsorption configurations of *o*X/*m*X in the structure of ZU-61 were constructed based on the experimental single-crystal X-ray diffraction data. Then, the single point energy calculations were performed to calculate the energy of these six possible configurations. All of the calculations were performed under the generalized gradient approximation (GGA) with Perdew-Burke-Ernzerhof (PBE) exchange correlation. The cutoff energy of 544 eV and 1×1×2 k-point mesh with smearing 0.1 ev were adopted in the calculation. Finally, the energy of these configurations was compared to confirm the stable *o*X/*m*X configuration in ZU-61 structure (Table 4). The higher negative value of single point energy corresponds to a more stable adsorption configuration.

Supplementary Table 1. Physical properties of C_8 aromatics[9,10]

Property	*p*-xylene	m-xylene	o-xylene	ethylbenzene
Molecular weight	106.17	106.17	106.17	106.17
Boiling point, °C	138.37	139.12	144.41	136.19
Kinetic diameter, Å	5.8	6.8	6.8	5.8
Dipole moment, $\times 10^{18}$ esu cm	0.1	0.37	0.64	0.59
Polarizability, $\times 10^{-25}$ cm^3	137-149	142	141-149	142

Supplementary Table 2. Summary of separation selectivity of C_8 aromatics in various MOFs[11,12]

	HKUST-1*	MIL-47[&]	MIL-125-NH_2[&]	MIL-140B[&]	MOF-48[&]	BaX[&]	ZU-61 This work
*m*X/*p*X	1.12	0.91	0.67	0.59	0.59	0.59	2.9

Supplementary Table 3. Crystal Structure data for ZU-61

Unit cell parameters	
Formula sum	C20 H18 F5 N4 Nb Ni O2
Formula weight	593 g/mol
Crystal system	tetragonal
Space-group	P 4/mmm
Cell parameters	a = b =11.2649(2) Å c = 7.8859(2) Å
Cell ratio	a/b=1.0000 b/c=1.4285 c/a=0.7000
Cell volume	1000.70(4) $Å^3$
Z	1

Supplementary Table 4. The single point energy of six possible adsorption configuration of *o*X and *m*X in ZU-61

Configuration	*o*X energy (ev)	*m*X energy (ev)
1	-23608.51676	-12505.51121
2	-23609.03129	-12505.5541
3	-23608.17046	-12505.49799
4	-23608.60259	-12505.51339
5	-23608.1285	-12505.56024
6	-23608.13254	-12505.52694

Supplementary Table 5. Relative Quality Correction Factor of p-X, m-X, o-X, and EB on FID

Component (*i*)	p-xylene	m-xylene	o-xylene	ethylbenzene
f_i	1	1.017	1.011	1.002

Reference chemical: p-xylene

Supplementary Table 6. A, B, C value of *p*X, *m*X, *o*X, and ethylbenzene

Compound	A	B	C
*p*X	6.99052	1453.430	215.307
*m*X	7.00908	1462.266	215.105
*o*X	6.99891	1474.679	213.686
EB	6.95719	1424.255	213.206

Supplementary Table 7. The corresponding relationship between each xylene isomer concentration and the response value of FID detector

Compound	Partial Pressure kPa	Area (FID)
*p*X	0.276	4490553
*m*X	0.272	4403553
*o*X	0.27	4475102
EB	0.27	4566984

Liquid composition: 5.0020 g EB, 5.3503 g *p*X, 5.5949 g *m*X, 6.9942 g *o*X

Supplementary Table 8. The binding energy of *p*X, *m*X, *o*X with ZU-61 calculated from SCXRD and DFT.

	*p*X (I)	*p*X (II)	*m*X	*o*X
Binding energy (kJ/mol)	72.9	62.7	50.1	109.8

Supplementary Table 9. The adsorption enthalpy of *p*X, *m*X, *o*X with ZU-61 measured by TG-DSC

	*p*X	*m*X	*o*X
Adsorption enthalpies (kJ/mol)	78	82	92

Supplementary Table 10. Summary of the amount of xylene isomers captured from breakthrough experiments at 333 K

	*p*X	*m*X	*o*X
Uptake Capacity mmol/g	0.62	1.15	1.61

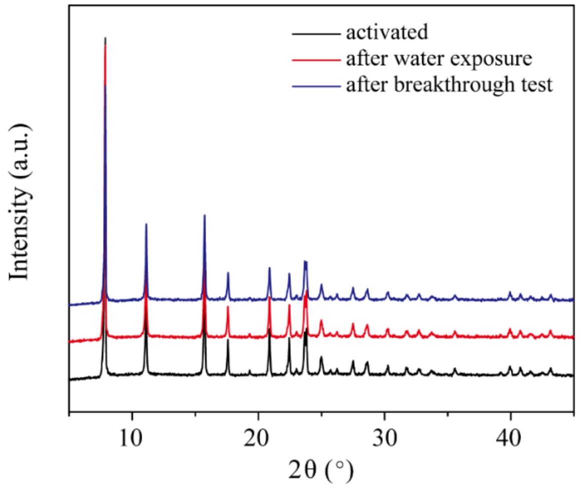

Supplementary Figure 1. The powder x-ray diffraction tests of ZU-61

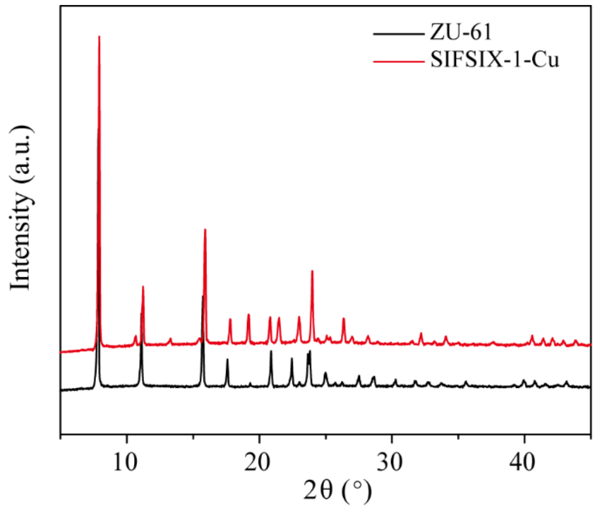

Supplementary Figure 2. Comparison of the powder x-ray diffraction tests of ZU-61 and SIFSIX-1-Cu

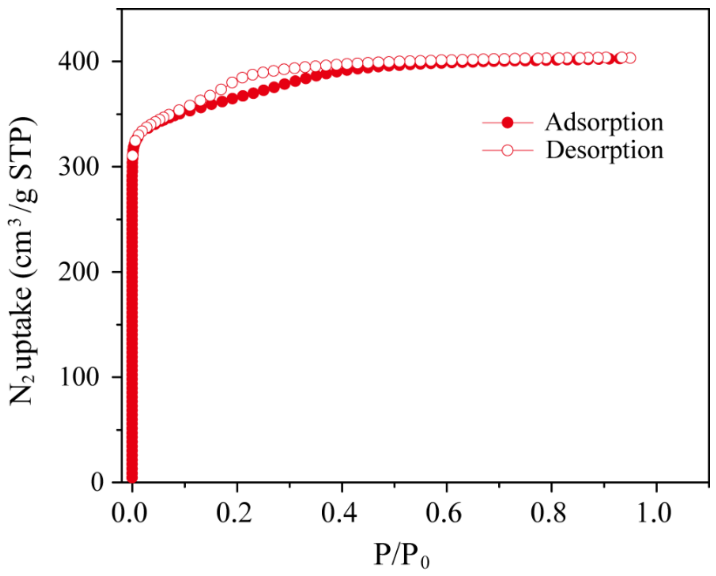

Supplementary Figure 3. N_2 adsorption-desorption isotherms of ZU-61 at 77K

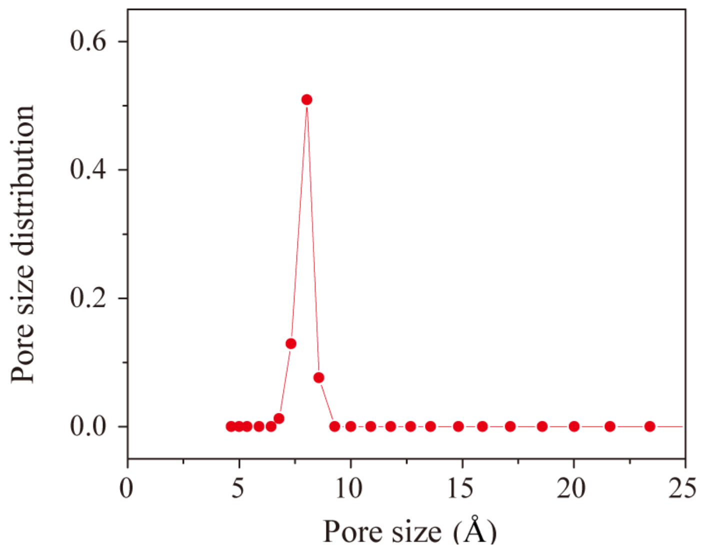

Supplementary Figure 4. Pore size distribution of ZU-61

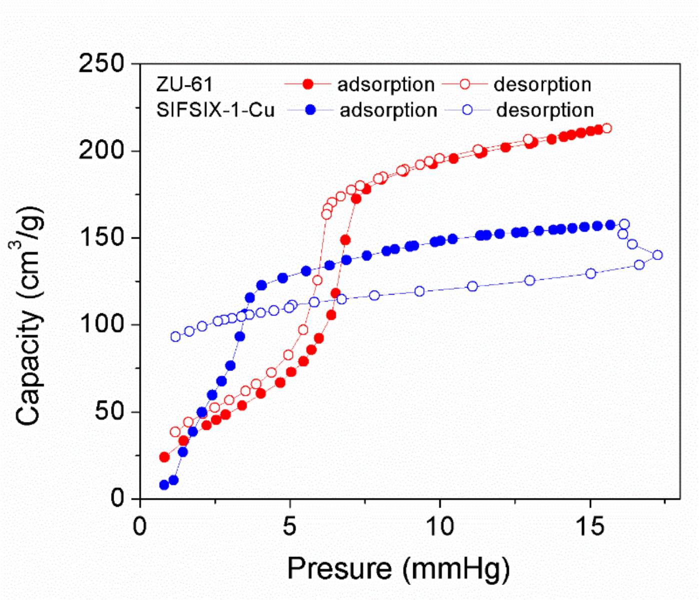

Supplementary Figure 5. Water adsorption isotherms of ZU-61 and SIFSIX-1-Cu at 298 K

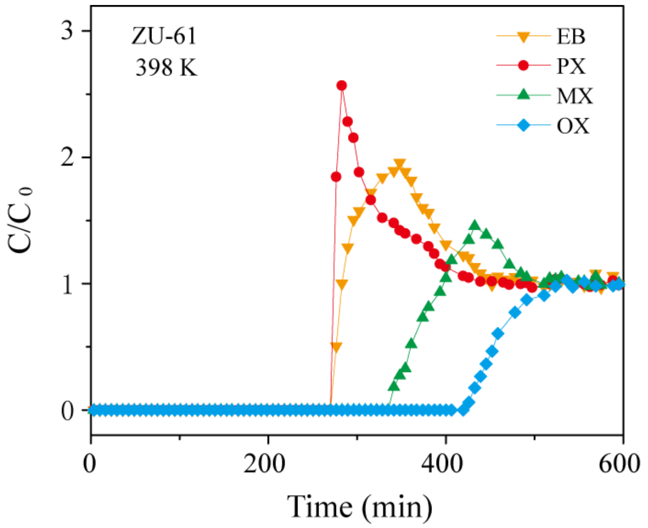

Supplementary Figure 6. Experimental column breakthrough curves for 1:1:1:1 *p*X/*m*X/*o*X/EB separations with ZU-61 at 398 K

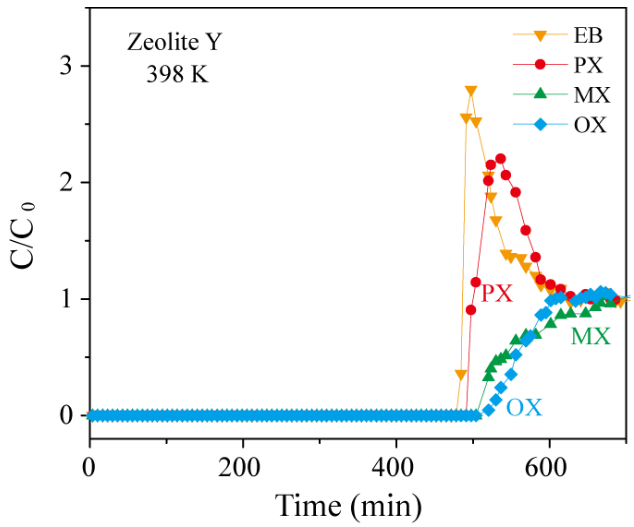

Supplementary Figure 7. Experimental column breakthrough curves for 1:1:1:1 *p*X/*m*X/*o*X/EB separations with zeolite NaY at 398 K

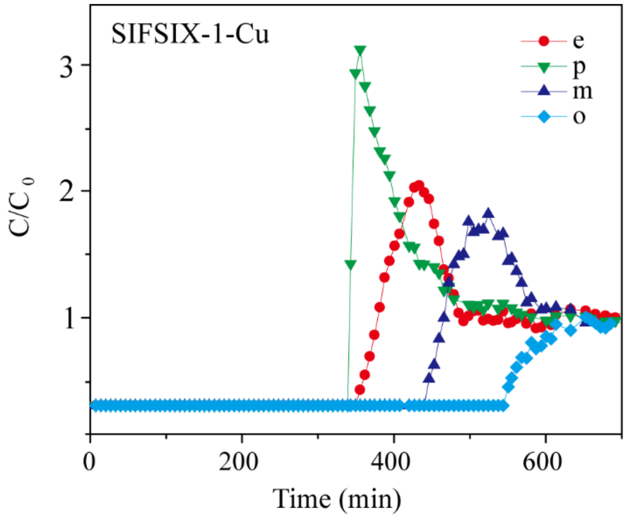

Supplementary Figure 8. Experimental column breakthrough curves for 1:1:1:1 *p*X/*m*X/*o*X/EB separations with SIFSIX-1-Cu at 398 K

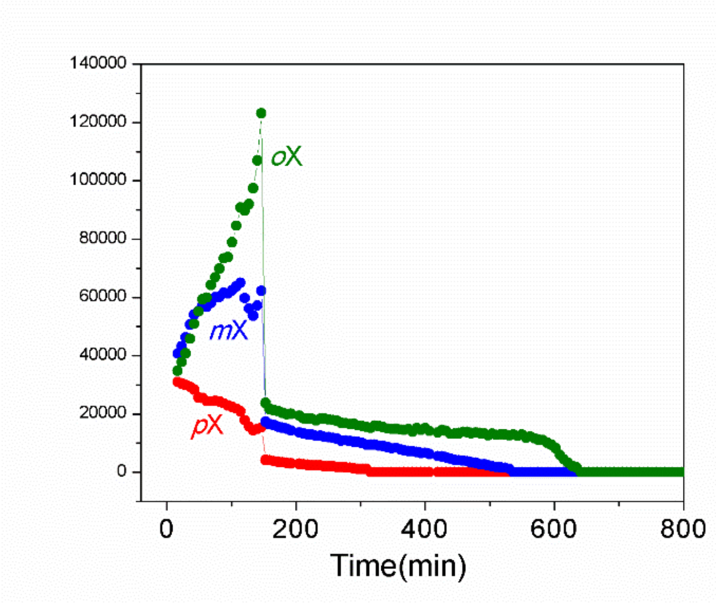

Supplementary Figure 9. Regeneration of ZU-61 after multi-component breakthrough experiments

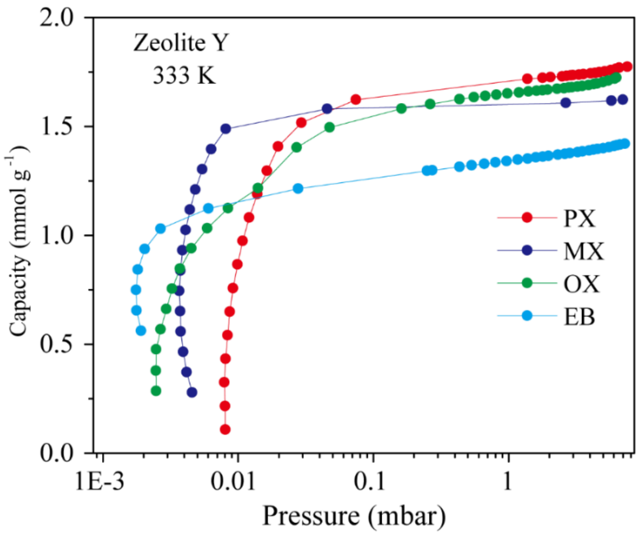

Supplementary Figure 10. The comparison of *p*X, *m*X, *o*X, and EB on zeolite NaY at 333 K

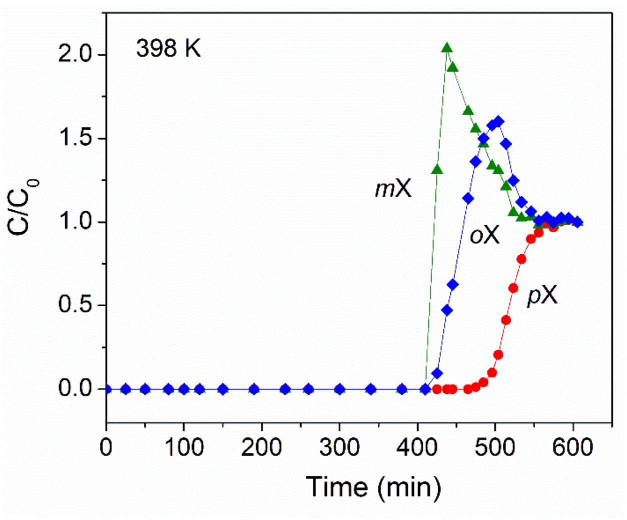

Supplementary Figure 11. Gas-phase breakthrough curves for 1:1:1 *p*X/*m*X/*o*X separations with BaY

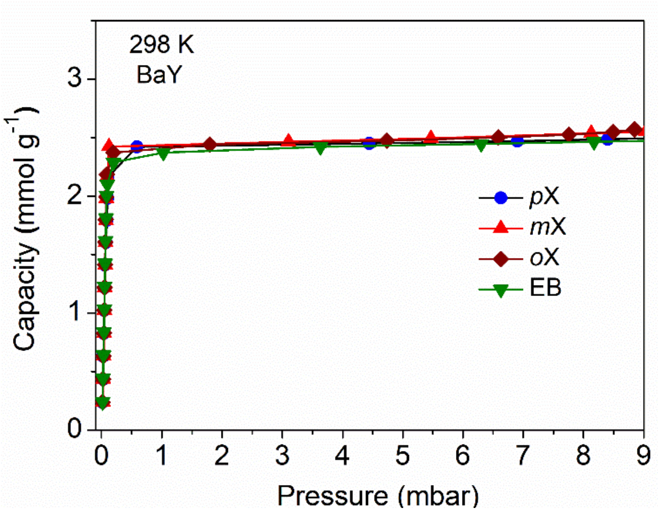

Supplementary Figure 12. Adsorption isotherms of *p*X, *m*X, *o*X, and EB on zeolite BaY at 298 K

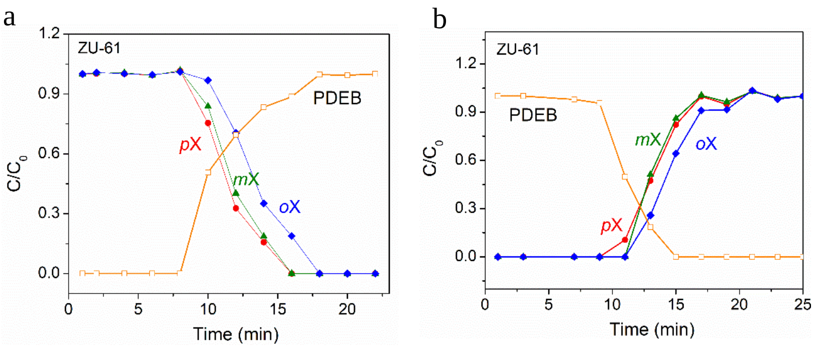

Supplementary Figure 13. Liquid-phase breakthrough experiments on zeolite ZU-61. Desorption curves by washed with PDEB at 298 K (a), and then the second cycle of the breakthrough curves (b). Note: the xylene mixtures were diluted with heptane and each xylene concentration in heptane is 0.01 mmol/mL.

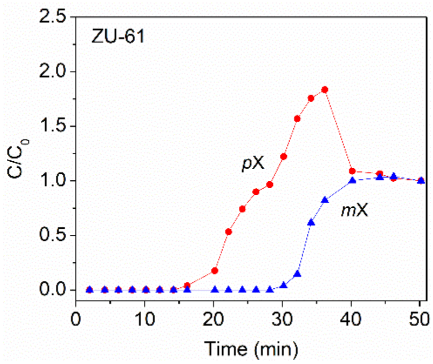

Supplementary Figure 14. Breakthrough curves for 1:1 pX/mX performed on ZU-61 at 298 K. Note: the xylene mixtures were diluted with hexane and each xylene concentration in hexane is 0.01 mmol/mL.

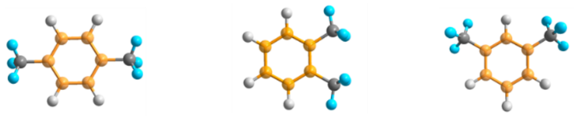

Supplementary Figure 15. The molecular structure of *p*X, *m*X, and *o*X. It should be note that there are two kinds of hydrogen in xylene molecules: aromatic hydrogen (H_{aryl}) that bind to the benzene ring and methyl hydrogen (H_{benzyl}, highlighted in blue).

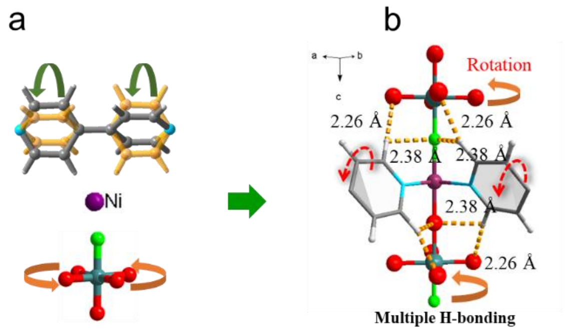

Supplementary Figure 16. Schematic illustration of the construction of ZU-61with of rotational $NbOF_5^{2-}$ anion, bipyridine (a), the intraframework interactions between anions, metal ion, and organic linkers (b).

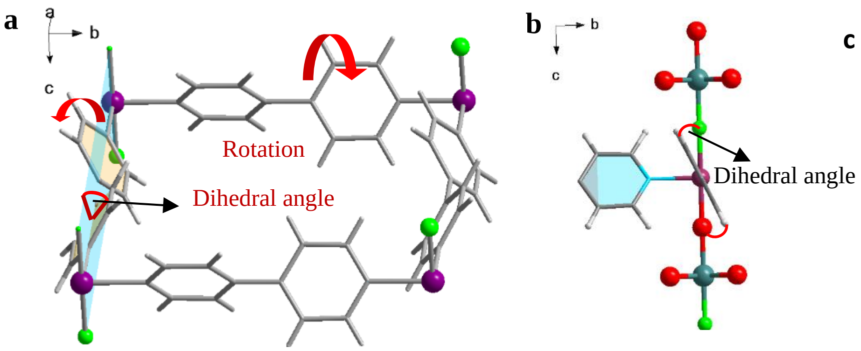

c

	Dihedral angle
ZU-61	34.06°
ZU-61·*p*X	26.17°
ZU-61·*m*X	25.86°

Supplementary Figure 17. The rotations (a) of organic ligand before and after the adsorption of xylene molecules, the dihedral angel (b), and the values of dihedral angel (c) of ZU-61·*p*X and ZU-61·*m*X.

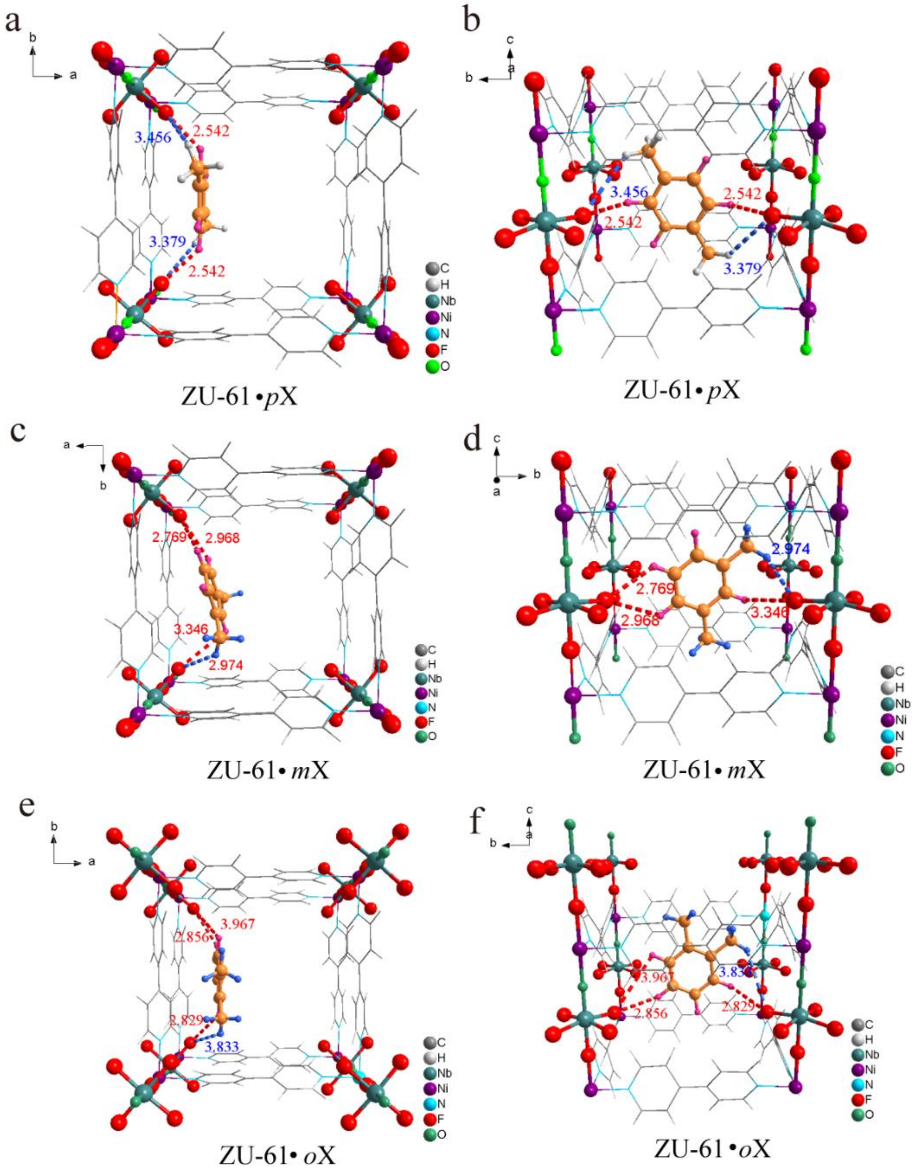

Supplementary Figure 18. DFT-D-simulated optimized xylene isomer adsorption sites of ZU-61. Adsorption positions of *p*X (a, b), *m*X (c, d), *o*X (e, f) molecules in the unite cell of ZU-61. Color code: C, gray-50%; H, gray-10%; Nb, teal; Ni, violet; O, green; N, sky blue; F, red.

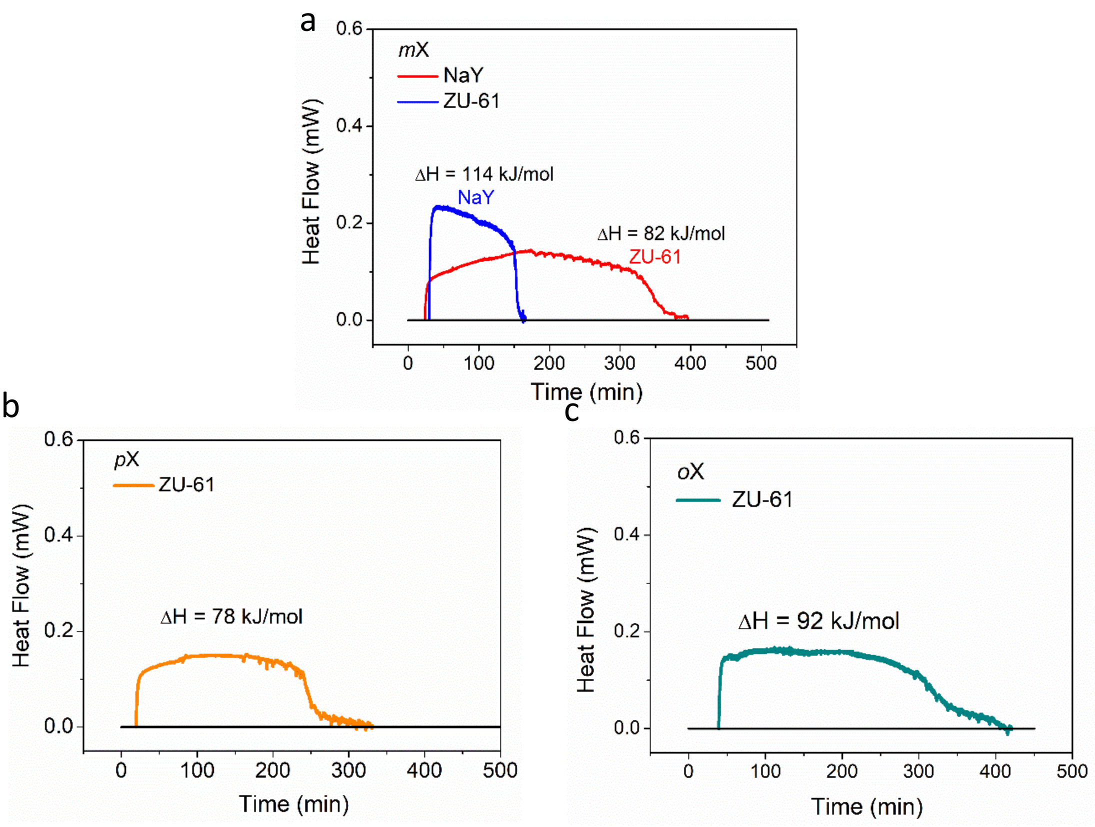

Supplementary Figure 19. Date of TG-DSC measurements on activated ZU-61 and zeolite NaY with *m*X (a), *p*X (b), *o*X (c).

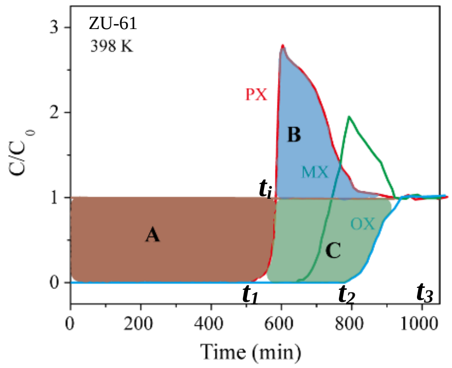

Supplementary Figure 20. The calculation for uptake capacity of *p*X and *o*X from the breakthrough curves

$$Q_{pX} = \frac{v{\cdot}n\%}{22.4{\cdot}m}(A-B) = \frac{v{\cdot}n\%}{22.4{\cdot}m}\{\underset{pX}{\int_0^{ti}}(C_0-C_i)dt - \left[\underset{pX}{\int_{t1}^{t3}} C_i dt - \underset{pX}{\int_{t1}^{ti}} C_i dt - \underset{pX}{\int_{ti}^{t3}} C_0 dt\right]\} \quad (2)$$

$$Q_{oX} = \frac{v{\cdot}n\%}{22.4{\cdot}m}(A+C) = \frac{v{\cdot}n\%}{22.4{\cdot}m}\underset{oX}{\int_0^{t3}}(C_0-C_i)dt \quad (3)$$

v, the flow rate, ml/min; *n*%, volume content; *m*, weight of the adsorbent; *t*, time, min.

Reference

(1) Bruker. APEX3 (Version 2015.9). Bruker AXS Inc., Madison, Wisconsin, USA (2016).
(2) Bruker SAINT V8.35A. Data Reduction Software (2016).
(3) Sheldrick, G. M. SADABS. Program for Empirical Absorption Correction. University of Gottingen, Germany (1996).
(4) Sheldrick, G. M. "Crystal structure refinement with SHELXL", Acta Cryst. C71, 3-8 (2015).
(5) Sheldrick, G. M. Acta Cryst. A46, 467-473 (1990).
(6) Sheldrick, G. M. Acta Cryst. A64, 112-122 (2008).
(7) Sheldrick, G. M. Acta Cryst. A71, 3-8 (2015).
(8) Dolomanov, O. V.; Bourhis, L. J.; Gildea, R. J.; Howard, J. A. K. & Puschmann, H. OLEX2: A complete structure solution, refinement and analysis program. J. Appl. Cryst. 42, 339-341 (2009).
(9) Cannella, W. J. Xylenes and Ethylbenzene. Kirk-Othmer Encyclopedia of Chemical Technolog; John Wiley & Sons: New York, **2000**.
(10) Bao, Z. B.; Chang, G. G.; Xing, H. B.; Krishna, R.; Ren, Q. L.; Chen, B. L. *Energy Environ. Sci.*, **2016**, 9, 3612-3641.
(11) Peralta, D.; Barthelet, K.; Pérez-Pellitero, J.; Chizallet, C.; Chaplais, G.; Simon-Masseron, A.; Pirngruber, G. D. *J. Phys. Chem. C* **2012**, 116, 21844−21855.
(12) Gee, J. A.; Zhang, K.; Bhattacharyya, S.; Bentley, J.; Rungta, M.; Abichandani, J. S.; Sholl, D. S.; Nair, S. *J. Phys. Chem. C* **2016**, 120, 12075-12082.
